# Physical abuse of young children reported by medical professionals in the United States 2014–2023

**DOI:** 10.3389/fped.2025.1718486

**Published:** 2026-01-05

**Authors:** Holly Hughes Garza, Karen E. Piper, Karla A. Lawson

**Affiliations:** 1 Trauma and Injury Research Center, Dell Children’s Medical Center, Trauma Services, Austin, TX, United States; 2 Department of Surgery and Perioperative Care, Dell Medical School, University of Texas at Austin, Austin, TX, United States

**Keywords:** age factors, child, infant, physical abuse, United States

## Abstract

**Background:**

Medical professionals play an important role in identifying suspected physical abuse of young children, who are at higher risk of serious or fatal abuse, and reporting it to child protection agencies. A recent publication suggested the rate of investigations of physical abuse in infants stemming from medical professionals' reports may be increasing.

**Objectives:**

To evaluate trends in rates of investigation of physical abuse concerns involving children under 5 years of age reported by medical professionals in the United States from 2014 to 2023, within the context of concurrent changes to national reporting rules, and to examine rates of substantiation of such reports, and resulting rates of entry into foster care.

**Methods:**

The National Child Abuse and Neglect Data System Child Files were used to identify investigated reports for this cohort study. Four states showing abrupt increases in investigations of physical abuse coinciding with changes in national reporting were excluded. Age-specific rates of investigation, substantiation, and foster care entry were calculated, and trend over time was tested using a Mann Kendall trend test. A generalized linear mixed effects model was used to estimate the odds of case substantiation, adjusting for child's age, sex, race/ethnicity, other maltreatment reported, neighborhood conditions, and random effects of state or territory.

**Results:**

Of 285,329 report-child pairs with physical abuse concerns reported by a medical professional, 51% were infants under 1 year of age (*n* = 146,518). Physical abuse was substantiated in 31% of 284,610 cases with a determination available (*n* = 86,977), 50% of 237,688 available cases received any type of post-investigation services (*n* = 119,015), and 18% of 213,986 available cases entered foster care as a result of the investigation (*n* = 37,698). Population-adjusted investigation rates did not change significantly over the 10 years (*p* = 0.72). Infants had the highest adjusted odds of substantiation (aOR 2.63, 99% CI 2.57–2.69 vs. children 1–4 years of age). Misclassification of infant prenatal substance exposure as physical abuse presented a significant challenge in assessing trends.

**Conclusions:**

This study suggests rates of investigations of physical abuse concerns involving children under 5 years of age reported by medical professionals in the United States have not significantly increased in recent years.

## 1 Introduction

Physical child abuse is a preventable global public health problem that is underreported to child protection agencies (1). Definitions of physical child abuse vary and change over time, making it difficult to compare incidence and trends between countries or between states in the US (1, 2). In the United States (US), physical abuse includes “physical acts that caused or could have caused physical injury to a child”, including “excessive or unreasonable corporal punishment.” (3) Children under 5 years of age experience serious physical abuse at higher rates than older children in the US, and in particular, infants are more likely to die from physical abuse than any other age group (3). In 2023, 24 infants per 100,000 US population died from child maltreatment, with 42% of those involving physical abuse (3). Medical professionals are one of the most frequent reporters of suspected child maltreatment to Child Protective Services (CPS) agencies in the US (3, 4). Two recent studies showed increasing investigations from reports to US CPS agencies by medical professionals, particularly those involving children younger than 1 year of age (4, 5). Expansion of national reporting requirements for “infants with prenatal substance exposure (IPSE)” has been suggested as a key driver of this apparent increase (4).

The Federal Comprehensive Addiction and Recovery Act of 2016 (CARA) required US states to begin reporting infants “born with and identified as being affected by substance abuse or withdrawal symptoms resulting from prenatal drug exposure, or a Fetal Alcohol Spectrum Disorder” to the National Child Abuse and Neglect Data System (NCANDS), and states are advised to incorporate these reports under the category of neglect (not physical abuse) that is reported by a medical professional (3, 6, 7).

A limitation of existing research on child maltreatment investigation rates using NCANDS data is the incomplete disaggregation of investigations by child age group, profession of the reporter, and maltreatment type (e.g., physical abuse vs. neglect) (5, 8, 9). Furthermore, rates of substantiation of reports by CPS agencies, and rates of resulting placement outside the home seem to vary by reporter type (professional vs. non-professional, and between professions such as law enforcement, medical, and education), but few studies on this topic have examined specific maltreatment types within single reporter types (5, 8, 9).

To address this gap in the current literature, we analyzed the NCANDS Child Files, FFY 2013–2023, using the subset of investigations resulting from a medical professional's report involving children under 5 years of age with concern for physical abuse, stratified by age subgroup (<1 year, 1–2 years, and 3–4 years). Trends in investigation rates over time in this population were evaluated, as well as outcomes (CPS substantiation of the report, and foster care entry) and bivariate and multivariable associations of demographic and other covariates with the odds of report substantiation. We hypothesized that infants would have higher adjusted odds of substantiation for physical abuse than older children.

## 2 Methods

### 2.1 Data sources

Data were obtained from the NCANDS Child File datasets regarding investigations by state CPS agencies in the United States (10). NCANDS is the most comprehensive dataset of child maltreatment reported to CPS agencies in the US; all 50 states and the District of Columbia submit data, including case-level data on investigated reports (3). Roughly half of all reports made to CPS agencies are “screened out” by the agencies: no investigation occurs in these cases (3). Investigations result in a “substantiated” disposition if the maltreatment “is supported or founded by state law or policy” (3). These files include administrative data on reports of possible maltreatment with completed investigations from CPS agencies in all 50 US states, the District of Columbia, and Puerto Rico (3). However, NCANDS is not a true population-based registry such as those available in some European countries (11). Reports in this dataset are only those that were “screened in” by CPS agencies—meaning they assessed the report as meeting state-specific criteria to move forward either to an investigation or some type of Alternative Response (3). Data for the Child Files are collected by the National Data Archive on Child Abuse and Neglect (NDACAN), a service of the Children's Bureau, U.S. Department of Health & Human Services. NCANDS data undergoes mapping for variable uniformity across states, data validation, and technical review, but final data quality varies between states, with some noted to have duplicates and a few states using dummy IDs for unidentified children (7). NCANDS Child Files are organized by Federal Fiscal Year (October 1 of the previous year to September 30; hereafter “study year”) as determined by the date of final disposition of the report to CPS. States may submit updates if a report disposition changes, which could occur in a subsequent study year (7).

Annual and state-specific population estimates from the U.S. Census Bureau were obtained by single-year age using CDC WONDER (12). Child Opportunity Index (COI) 3.0 scores were obtained from diversitydatakids.org, based at the Institute for Equity in Child Opportunity & Healthy Development at Boston University School of Social Work (13). This index is based on 44 indicators in the domains of education, health and environmental settings, and socioeconomic conditions.

### 2.2 Study cohort

**Figure 1:**
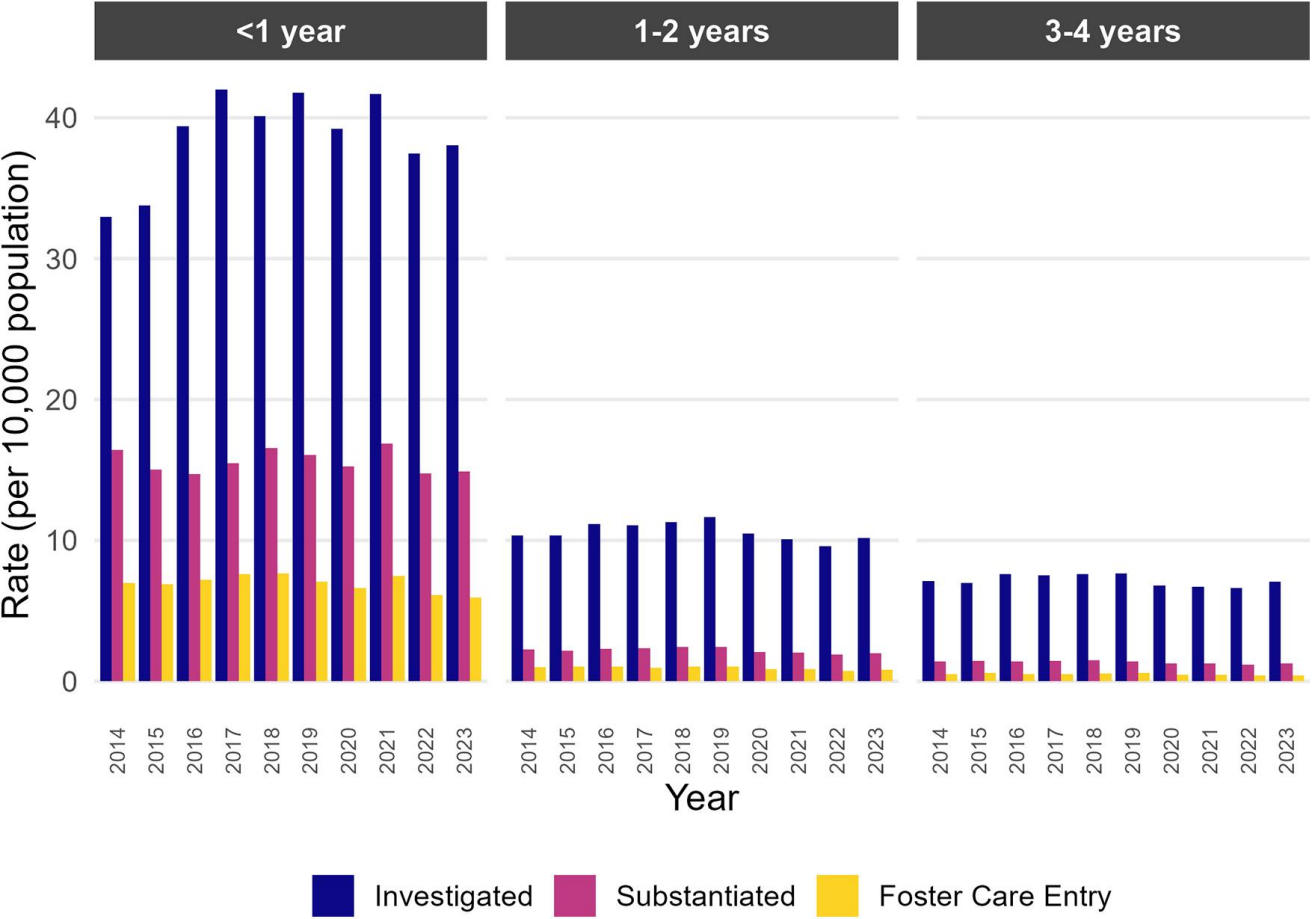
Physical abuse investigations stemming from CPS reports by medical professionals involving “infants with prenatal substance exposure,” by state and year, 2014–2023.

This cohort study included NCANDS Child Files from study years 2014 through 2023 appended into a single dataset. Because a single CPS report may involve multiple children, or a child may be involved in multiple reports, the report-child pair (combined with state identifier to ensure uniqueness) was used as the level of analysis (hereafter referred to as “investigations”). Any duplicate report-child pairs were removed in accordance with NDACAN guidance (7), keeping the record with the most recent report disposition. Investigations were then included in the study cohort if the child's age at the time of the CPS report was less than 5 years, the report involved physical abuse of that child, and the report source was a medical professional (*n* = 413,554). Records were excluded if the record was from Puerto Rico (*n* = 464; due to limitations in population data). Temporal trends in investigations were visually evaluated by state (Figure 1) and any state that showed a sudden increase in physical abuse investigations between 2016 and 2018 (coinciding with changes in IPSE reporting) were excluded from the analysis. Investigations from the states of West Virginia, Michigan, Ohio, and Nevada were thus excluded from the main analyses.

### 2.3 Measures

Demographic variables were obtained from the NCANDS Child Files, for each case, including the child's age, sex, race, and ethnicity along with their county of residence and its urbanicity. Three age groups were created (<1, 1–2, and 3–4 years of age), grouping ages that had similar incidence rates together. A combined variable was created based on the reported race and ethnicity of the child, based on 5 binary variables provided in the datasets for race and 1 binary variable for Hispanic ethnicity. A single race/ethnicity was assigned to each report-child pair using the following sequence: Hispanic, non-Hispanic Black or African American (NH Black), non-Hispanic Asian (NH Asian), non-Hispanic American Indian or Alaska Native (NH AIAN), non-Hispanic Native Hawaiian or Other Pacific Islander (NH NHPI), or non-Hispanic White (NH White). Rural Urban Continuum Codes were re-categorized from the 9 categories provided with the NCANDS Child Files to 3 categories: Metropolitan (Codes 1–3), Non-Metropolitan Urban (Codes 4–7), and Rural (Codes 8–9).

Several types of child maltreatment are included in the NCANDS Child Files, which are defined primarily by state laws and policies. Although this study was restricted to investigations with reported physical abuse, each child may have documentation of up to four types of reported maltreatment on the same report. NCANDS guidance notes that “risk of physical abuse” may be coded as either physical abuse or emotional maltreatment. IPSE is a designation assigned by NDACAN when a child is (i) less than one year of age, (ii) the report source was a medical professional, and (iii) there was reported prenatal exposure to alcohol or drugs. The definition of this variable does not detail which specific drugs should be included. NDACAN advises states to categorize fetal alcohol syndrome or prenatal substance exposure as neglect when submitting reports to NCANDS, not physical abuse.

Each investigation is assigned up to 5 dispositions, or levels of finding as a result of CPS investigation. One disposition is assigned to the report as a whole and one for each of up to four maltreatment types for each child. We utilized the disposition corresponding to the reported physical abuse. Disposition options vary between states, for example some states utilize an “alternative response” rather than a traditional CPS investigation for certain reports. Eligibility for alternative response (AR) is unique to each state but AR generally indicates that the immediate risk to the child was not deemed high and the family voluntarily accepted some type of services from CPS (3). Dispositions of “substantiated,” “indicated,” or “reason to suspect,” are considered indications of likely maltreatment (hereafter “substantiated”), although the specific use of these terms varies between states (3). We utilized the NCANDS variable of “prior victim” of maltreatment which indicates there was a previous substantiated or indicated maltreatment disposition, though this designation is not always specific to physical abuse.

Variables related to child and caregiver characteristics were missing in large proportions (Supplementary Table S1). Because about 40% of data on individual socioeconomic status (SES) is missing in NCANDS, COI was utilized as an alternative measure of geographic advantage/disadvantage as suggested in a recent discussion of this NCANDS limitation (14). Small counties are de-identified in NCANDS to protect privacy, resulting in some missingness of COI but there was more available data on COI than any of the individual SES variables. COI 3.0 is a composite index of neighborhood-level conditions that impact a child's healthy development and access to opportunities as an adult (13). Because the most granular unit of geography in NCANDS Child Files is county, and COI can vary substantially within a county, we chose the “Within-County Child Opportunity Levels” data file (15). This allowed us to join the overall proportion of children living in a “Very Low” opportunity neighborhood to the county of each report-child pair, as a measure of the level of concentrated disadvantage where the child lives. These proportions were then categorized into quartiles, where the highest proportion of children in “Very Low” COI neighborhoods corresponds to the most concentrated disadvantage.

Services or activities directly related to the CPS response and delivered within 90 days after the disposition date are categorized as “post-investigative services.” Examples broadly include family support services, family preservation services, certain court actions, case management, counseling, education, home health, housing assistance, family planning, and other service referrals. Foster care is included as a post-investigative service if the child is in foster care for more than 24 h at any time from the date of the CPS report to 90 days after the case disposition date, and includes relatives if the child is placed in their care as part of the CPS investigation. Approximately 80% of child maltreatment deaths are included in the Child Files, the remainder are reported to NCANDS only in aggregate, and the state/territory is masked to protect privacy by NDACAN when the report involves a fatality (3, 7). We included investigations that involved a child death, but did not report fatality rates because not all fatalities are represented.

### 2.4 Analysis

Analysis was conducted using the R programming language [vers. 2024.04.1 (16),]. Age-specific rates were calculated for each study year by dividing the number of unique report-child pairs by the estimated population of children in each single-year age or age group. Child, caregiver, and other demographic characteristics were compared by age group using chi-square tests. A generalized linear mixed effects model with random effects by state/territory was used to assess the odds of a report being substantiated, by age group and other covariates. Model fit was performed using maximum likelihood (Laplace Approximation) with Wald confidence intervals by means of the lme4 package [vers. 1.1–37 (17)]. Covariates were chosen for inclusion in the model based on prior research on relevant confounders. Urbanicity was initially included but was removed based on a lower Akaike Information Criterion (AIC) value of the nested model without urbanicity. Tests for trend in investigation rates in each age group were conducted using the Mann Kendall trend test with a 2-sided test of significance. The ggplot2 package was used to visualize results [vers. 3.5.1 (18)]. A *p*-value of <0.01 was considered statistically significant for all hypothesis testing. Complete case analysis was utilized and missing data was not imputed. Because 20% of investigations were missing data on COI, due to masking of some counties in the Child Files, we conducted a sensitivity analysis without using any measure of SES in the multivariable analysis. The resulting model had the benefit of larger sample size, but showed inferior model fit. We utilized the STROBE (Strengthening the Reporting of Observational studies in Epidemiology) Guidelines for reporting observational studies.

The institutional review board at the study institution determined that this research meets the criteria for exemption from review as secondary research on data or specimens. Written informed consent for participation was not required from the participants or the participants' legal guardians or next of kin in accordance with national and institutional requirements.

## 3 Results

### 3.1 Descriptive statistics

From 2014 to 2023 there were 413,090 investigations of physical abuse involving children less than 5 years of age for which the report source was a medical professional. After exclusion of investigations from West Virginia, Michigan, Ohio, and Nevada, the main analysis included 285,329 investigations. The median age of these children was <1 year (IQR 0–2 years). Among investigations involving children less than 5 years of age reported to CPS agencies by medical professionals with physical abuse concerns, over half were infants (<1 year of age), and male children were reported more often than females regardless of age group (Table 1). Investigated reports involved a young child with a prior substantiated maltreatment report 10% of the time overall, and the proportion of children with a prior substantiated report was significantly higher in older age subgroups compared with infants (*p* < 0.01). There were statistically significant differences across age subgroups in all demographic variables examined (Table 1).

**Table 1:** Characteristics of children less than 5 years of age reported to CPS agencies by medical professionals with physical abuse concerns in 2014–2023.

Characteristics	Overall	<1 year	1–2 years	3–4 years
	*N* = 285,329^1^	*N* = 146,518^1^	*N* = 82,086^1^	*N* = 56,725^1^
Sex^a^^,^^b^				
Female	127,587 (45%)	66,733 (46%)	36,174 (44%)	24,680 (44%)
Male	156,413 (55%)	79,139 (54%)	45,473 (56%)	31,801 (56%)
Race/ethnicity^a^^,^^b^				
NH White	118,695 (47%)	59,009 (47%)	34,782 (47%)	24,904 (48%)
NH Black	69,646 (28%)	36,175 (29%)	20,012 (27%)	13,459 (26%)
Hispanic	55,691 (22%)	27,198 (22%)	16,788 (23%)	11,705 (23%)
NH Asian	4,033 (1.6%)	1,785 (1.4%)	1,322 (1.8%)	926 (1.8%)
NH AIAN/NHPI	4,674 (1.8%)	2,201 (1.7%)	1,463 (2.0%)	1,010 (1.9%)
Urban/rural^a^^,^^b^				
Metro	232,315 (82%)	119,155 (82%)	66,817 (82%)	46,343 (82%)
Non-metro	43,463 (15%)	22,335 (15%)	12,617 (15%)	8,511 (15%)
Rural	7,821 (2.8%)	4,199 (2.9%)	2,089 (2.6%)	1,533 (2.7%)
Concentrated disadvantage^a^^,^^b^^,^^c^				
Q1 (Lowest)	56,833 (25%)	27,861 (24%)	16,916 (26%)	12,056 (27%)
Q2	56,833 (25%)	27,785 (24%)	16,993 (26%)	12,055 (27%)
Q3	56,832 (25%)	29,353 (25%)	16,484 (25%)	10,995 (24%)
Q4 (Highest)	56,832 (25%)	31,764 (27%)	14,851 (23%)	10,217 (23%)
Prior victim^a^^,^^b^				
No	251,227 (90%)	138,505 (96%)	68,453 (86%)	44,269 (80%)
Yes	28,105 (10%)	5,735 (4.0%)	11,374 (14%)	10,996 (20%)
Other maltreatment on same report^a^				
Neglect	106,228 (37%)	53,405 (36%)	32,477 (40%)	20,346 (36%)
Medical neglect	9,708 (3.4%)	4,204 (2.9%)	3,608 (4.4%)	1,896 (3.3%)
Sexual abuse	4,520 (1.6%)	500 (0.3%)	1,575 (1.9%)	2,445 (4.3%)
Psychological abuse	4,786 (1.7%)	1,419 (1.0%)	1,771 (2.2%)	1,596 (2.8%)
Services received				
Post-investigation services^a^^,^^b^				
No	118,673 (50%)	52,266 (41%)	38,923 (59%)	27,484 (61%)
Yes	119,015 (50%)	74,091 (59%)	27,364 (41%)	17,560 (39%)
Foster care services^a^^,^^b^				
No	176,288 (82%)	89,726 (77%)	51,150 (87%)	35,412 (90%)
Yes	37,698 (18%)	26,452 (23%)	7,318 (13%)	3,928 (10.0%)

a Pearson's Chi-squared test <0.01.

b Percentages do not include missing data.

c Percentage of children living in quartile of counties with the largest proportion of Very Low opportunity neighborhoods from Child Opportunity Index 3.0. Includes 46 US states and the District of Columbia, excludes West Virginia, Michigan, Ohio and Nevada.

Drug abuse was the most commonly reported child risk factor (12% of investigations with available data; *n* = 19,991) and caregiver risk factor (19% of investigations with available data; *n* = 30,865). Of 146,518 investigations involving children under 1 year of age, IPSE was recorded in 21% as a combination of the risk factors of child alcohol (2.3%; *n* = 2,028) and drug (20%; *n* = 18,799) abuse (Supplementary Table S3).

Physical abuse concerns were substantiated by CPS in 31% of cases (*n* = 86,977). Only 83% of report-child pairs had data available on post-investigation services provided to the family. Overall, half of those with available data received any post-investigative services, and 18% entered foster care as a result of the investigation (Table 1). The proportion of investigations resulting in provision of any post-investigation services was highest for infants and decreased with age. The proportion of infants entering foster care was more than twice that of children 1 through 4 years of age (Table 1).

### 3.2 Population-based rates

**Figure 2:**
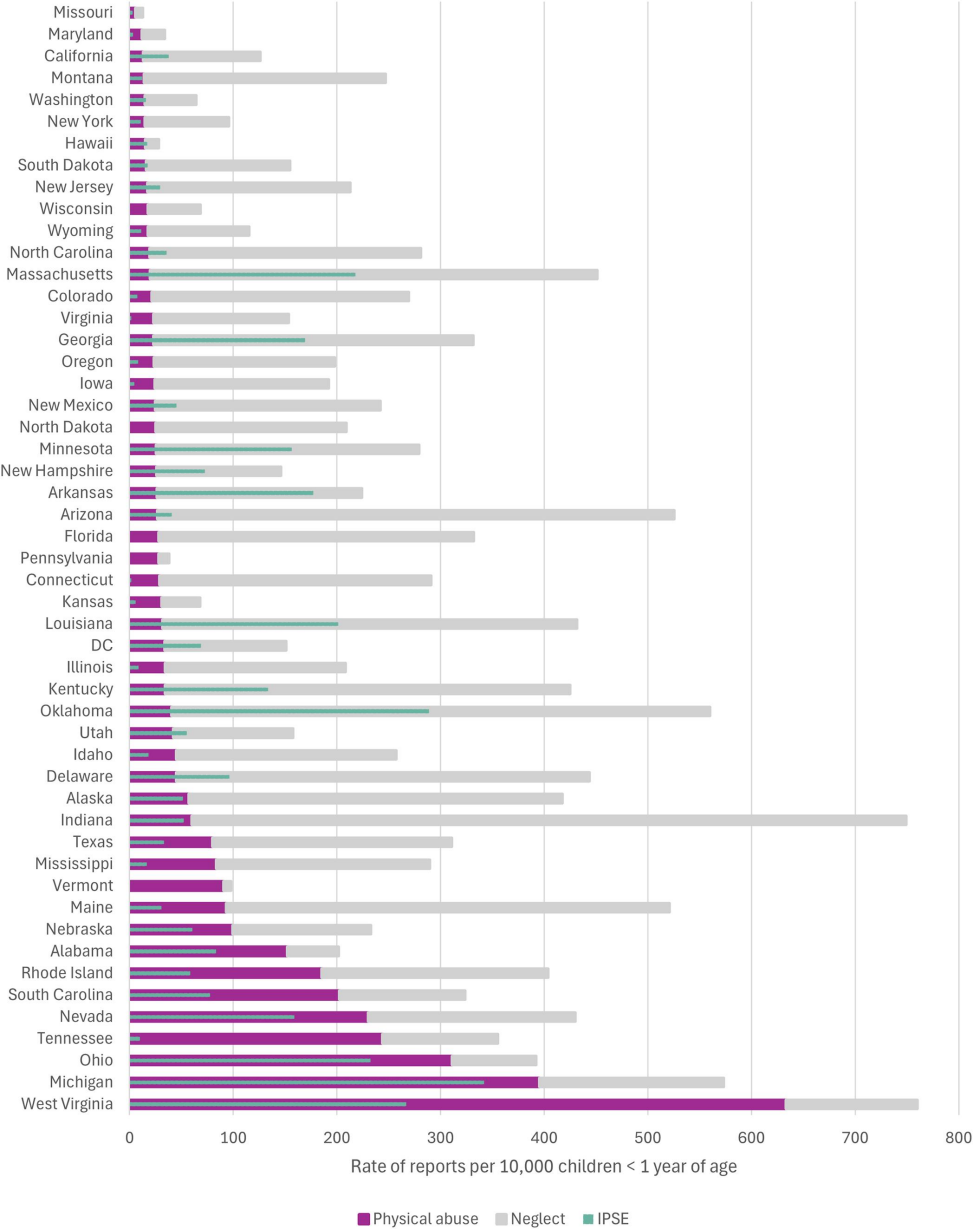
Age-specific population-based rates of investigation, substantiation of physical abuse, foster care entry, and death associated with physical abuse reports by medical professionals to CPS agencies involving children under 5 years of age (*n* = 285,329).

Age-specific rates of physical abuse investigations involving young children reported by medical professionals were consistently highest among infants (<1 year of age), (Figure 2). Between 33 and 42 investigations per 10,000 infants were noted annually, with rates of substantiated physical abuse ranging from 15 to 17 per 10,000 (Figure 2). Among children 1–4 years of age, fewer than 3 substantiated reports occurred per 10,000 (Figure 2). There was not a significant trend in the annual rate of investigations for infants (*p* = 0.72), 1–2-year-olds (*p* = 0.47), or 3–4-year-olds (*p* = 0.37) from 2014 to 2023. Annual foster care entry rates were 6.0–7.6 per 10,000 for infants, compared with infants compared to older children (0.6–1.0 per 10,000 for older children (Figure 2).

**Figure 3:**
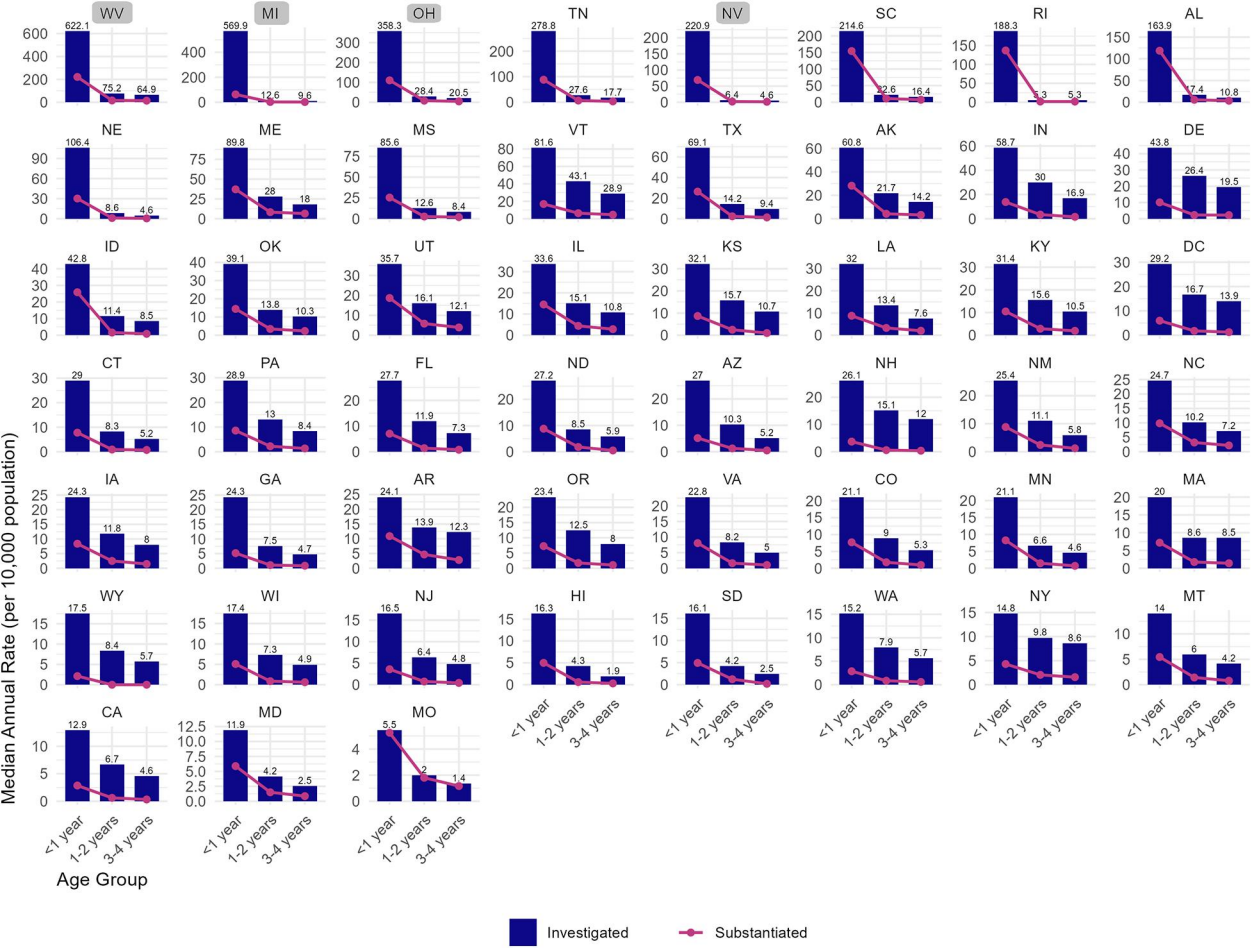
Variation by US state in the age-specific population-based rates of investigation and substantiation of physical abuse reported by medical professionals to CPS agencies involving children under 5 years of age, by age group.

Marked heterogeneity was noted in rates of investigation and substantiation of reports between states; of states included in the main analysis, the rate of investigations ranged from 5.7 per 10,000 in Missouri to 243.9 per 10,000 in Tennessee (Figure 3, Supplementary Table S2). While Tennessee had investigation rates of infants similar to that of the four states excluded from the main analysis (state abbreviations boxed in grey in Figure 3), the number of physical abuse investigations with IPSE in Tennessee did not surge notably (Figure 1).

### 3.3 Multivariable analysis

Substantiation of physical abuse reported by medical professionals was strongly associated with age, with infants having 2.66 times higher odds of substantiation compared to older children (aOR 2.66; 99% CI 2.58–2.74), after controlling for confounding factors. Substantiation was also significantly associated with having substantiated maltreatment on a previous report, having neglect on the same report as the physical abuse, and living in an area of concentrated disadvantage as measured by COI 3.0 (Table 2). Weaker associations with substantiation were noted for both sex and race/ethnicity, when adjusting for the other covariates. Male children had higher adjusted odds of substantiation than females. Children reported to be non-Hispanic Black had 14% higher adjusted odds of substantiation compared to non-Hispanic White children (aOR 1.14; 99% CI 1.10–1.18), while those reported to be non-Hispanic Asian children had 32% lower adjusted odds of substantiation than non-Hispanic White children (aOR 0.68; 99% CI 0.60–0.76). The adjusted odds of substantiation when the child was Hispanic or NH AIAN/NHPI were not significantly different from NH White using the predetermined *p*-value threshold of <0.01. Small differences were noted in the sensitivity analysis that removed any measure of SES (Supplementary Table S4). The only change in conclusions were than when SES was not considered, the adjusted odds of substantiation became significantly higher for NH AIAN/NHPI children compared to NH white children.

**Table 2:** Multivariable associations of child characteristics and adjusted odds of substantiation of a physical abuse report by a medical professional from 2014 to 2023 in children under 5 years of age.

Variable (fixed effects)	aOR	99% CI
Age		
1–4 years (Ref)		
<1 year	2.66	2.58, 2.74
Sex		
Female (Ref)		
Male	1.05	1.02, 1.08
Race/ethnicity­		
NH White (Ref)		
NH Black	1.14	1.10, 1.18
Hispanic	0.97	0.93, 1.00
NH Asian	0.68	0.60, 0.76
NH AIAN/NHPI	1.11	1.00, 1.25
Neglect also on report	1.43	1.38, 1.48
Prior victim	1.31	1.27, 1.35
Concentrated disadvantage^a^		
Q1 (Lowest % of children living in Very Low opportunity neighborhoods; Ref)		
Q2	1.04	1.00, 1.09
Q3	1.14	1.09, 1.19
Q4 (Highest % of children living in Very Low COI neighborhoods)	1.31	1.25, 1.36

a Based on within-county measure of Child Opportunity Index 3.0. Represents the percentage of children living in a Very Low Opportunity neighborhood for the child's county of residence.

Includes 46 US states and the District of Columbia, excludes West Virginia, Michigan, Ohio and Nevada.

*N* = 195,582, groups = 47.

Random effects: Adjusted ICC = 0.149.

AIC = 215,161.4.

### 3.4 IPSE and physical abuse reporting

**Figure 4:**
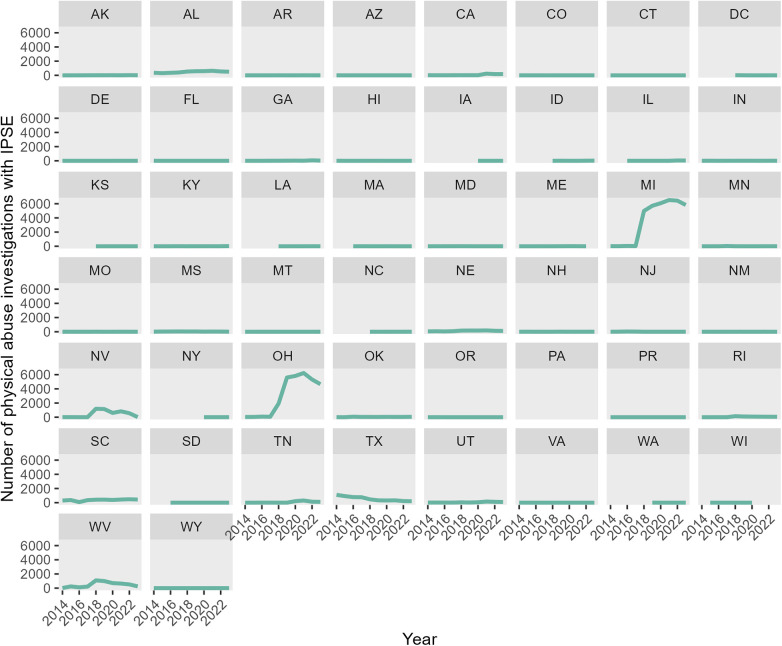
Population-based rate of investigations of possible physical abuse and neglect involving infants (<1 year of age) reported by medical professionals, and rate of reports with “infant prenatal substance exposure,” by US state from 2014 to 2023.

When looking at data from all 50 states and DC, 54% of infants (<1 year of age) with investigations of possible physical abuse reported by a medical professional were coded as IPSE (*n* = 93,952). Of those, 83% (*n* = 77,794) lacked a neglect allegation on the same report, suggesting deviation from NCANDS guidance to code IPSE-related reports as neglect. To better characterize this pattern, and its variation between states, we performed *post-hoc* analysis of physical abuse investigation rates in relation to investigation rates of neglect and IPSE among all infants reported by medical professionals (Figure 4). Differential use of neglect and physical abuse to categorize reports in this age subgroup was apparent. Four states (WV, MI, NV, and OH) were identified as having a combination of high rates of IPSE reporting, high reported rates of physical abuse, and abrupt increases in both IPSE and physical abuse reporting around 2017–2018. This timeframe coincided with the implementation of reporting changes from CARA.

## 4 Discussion

Overall, our findings did not show a clear time trend in investigations or substantiated cases of physical abuse reported by medical professionals of children under 5 years of age, including infants. Children in this age group are at high risk for serious injury or death from physical abuse (3), but little has been published on what happens to these specific reports by medical professionals once they enter the child welfare system. Our analysis showed that roughly one third of such investigated reports were substantiated, half received any type of services as a result of the investigation, and 18% resulted in foster care entry for the child. Further, multivariable analysis in this novel subpopulation of child maltreatment investigations showed that substantiation of screened-in physical abuse reports by medical professionals in children under 5 years of age was associated with the child's age, area-level disadvantage, and race/ethnicity. While we did not perform multivariable analysis for the outcomes of foster care entry, it was far more common among infants than other children younger than 5 years.

### 4.1 Results in context

While one study suggested an uptick in physical abuse reports by medical professionals in infants in the United States beginning in 2017 (4), our results indicate this may have been primarily an issue of report misclassification. It is difficult to find studies that calculate population-based rates from other countries that use a comparable study cohort and data source. A retrospective cohort study in France found a national incidence of physical abuse diagnosis in hospitals of 4 per 10,000 infants (19). This rate is considerably lower than our estimated rate of substantiated cases involving 15–17 per 10,000 infants, though the authors state that hospital data likely underestimates the overall incidence. It is unclear how this hospital-based estimate would compare to a study using child welfare administrative data similar to NCANDS. While our study aims did not include specifically looking for changes in rates related to the COVID-19 pandemic, our timeframe does include the pandemic-impacted years. Our finding of no significant change in investigation rates over time aligns with some other research indicating that physical abuse treated in hospitals did not change significantly during the pandemic (20).

Very few studies have explored trends in NCANDS data by report source. In 2023, medical professionals were the source of 11% of investigated child maltreatment reports in the United States, making them the third most common reporters overall after law enforcement and education personnel (3). However, medical professionals are more likely than other professionals to report maltreatment of younger children (5). Some evidence indicates that physical abuse is most likely to be substantiated when reported by a medical professional or law enforcement (5, 21). A 2010 study of military families found that 32%–26% of reports of physical child abuse with medical referral source were substantiated (*n* = 1,196 children of all ages) (9). One Canadian study found that maltreatment reports made by healthcare personnel were more likely to result in out-of-home placement than those made by other mandated reporters (22).

A study of NCANDS data that included maltreatment reports from the top 5 reporting professions (education, law enforcement, medical, social service, and mental health) from 2008 to 2018 found that 26% of investigated reports were substantiated and 12% resulted in foster care entry (5). Physical abuse reports were 1.2 times more likely to be substantiated and 1.6 times more likely to result in foster care entry than other maltreatment times in a multivariable analysis; similarly reports by medical professionals were 2.2 times more likely to be substantiated and 1.5 times more likely to result in foster care (5). Our findings align generally with the direction of associations noted in that study, which showed associations of younger age with both substantiation and foster care entry (5). Reasons for the observed variations in substantiation and out-of-home placements by CPS referral source may include training level of professionals on definitions of maltreatment, exposure of different professions with situations that pose more acute safety concerns, positioning to gather information about their concerns, perceived credibility of different professionals, and/or seriousness of maltreatment encountered (8).

The longer-term trajectory of children reported to CPS by medical professionals remains an area ripe for future research, including exploration of variation by maltreatment type and age group. If a reliable cohort of children can be identified from NCANDS data, then linkage between the Child Files and the Foster Care Analysis and Reporting System (AFCARS) files could allow longitudinal follow-up. One study utilizing such a linkage recently reported that children who remained in their homes after an initial report by a professional of any kind were less likely to have a subsequent CPS report more than 90 days later than those whose first report was from a non-professional (8). The authors hypothesized this might be because of differential “penetration” of these cases into the CPS system and subsequent provision of more family services that prevent repeated reports.

### 4.2 The need for better data

An unexpected takeaway from this study was that our ability to understand trends in CPS investigations of physical abuse of young children reported by medical professionals was hindered by changes in national data reporting. These changes were instituted in 2016–2018 in response to federal legislation (6, 7, 23). Despite guidance to report IPSE in NCANDS as neglect, some states now report physical abuse with IPSE at unusually high rates, as evidenced by high IPSE reporting rates and a surge in apparent physical abuse reporting by medical professionals in 2016–2018. The potential impact of this cannot be overstated. MI and OH data alone included reports of over 100,000 cases of physical abuse, roughly 25% of all such investigated reports nationwide.

These observations add to other recent explorations of potentially unintended consequences of changes in reporting mandated as part of CARA in 2016. Rebbe et al. in 2024 astutely pointed out the “fraught” methodology instituted by NDACAN to comply with this reporting change (23). By attempting to construct a variable for IPSE based on pre-existing variables of age <1 year, medical reporter source, and child alcohol or substance exposure, there is a risk of both underreporting IPSE (23) and conflating IPSE as a risk factor for abuse or neglect with IPSE as a reason for CPS reporting, in and of itself. In fact, Michigan's policy that “physical abuse includes drug or alcohol exposed infants” is documented in the State Child Abuse and Neglect (SCAN) Policies Database (24). Unfortunately, this practice coupled with NDACAN's broad use of age <1 year to define IPSE obscures the ascertainment of cases of true physical abuse in infants which often occurs months after delivery and can be serious or fatal.

The term “infants with prenatal substance exposure” itself in the national child maltreatment dataset seems to be inconsistently used. CARA specified inclusion in NCANDS of “substance abuse or withdrawal symptoms resulting from prenatal drug exposure, or a Fetal Alcohol Spectrum Disorder.” Not all infants who were exposed to alcohol or drugs prenatally go on to develop symptoms of withdrawal (25, 26). This distinction and the use of different medical terms in public health reporting is well-described in a 2020 paper using data from Massachusetts, where the state has required collection of data on these diagnoses by state public health authorities since 2016 (27). States vary in their criteria for prenatal substance exposure as it relates to the child welfare system (28). Developing a system to more consistently collect national data on substance use disorders and their effects on children in the context of the child welfare system may best be accomplished through collaboration with epidemiologists and other public health professionals who specialize in medical definitions related to prenatal substance exposure and its consequences.

In the meantime, there is currently insufficient information in the NCANDS Child Files to differentiate newborns reported to NCANDS only due to IPSE from infants with IPSE reported for physical abuse. NDACAN could mitigate the problem by adding an age category for children who were newborns (e.g., less than 15 days of age) at the time of the CPS report, as detailed by Rebbe et al. (23). Given our findings, this should be made available in the Child Files for all children, not just those with reported IPSE as it is currently defined in NCANDS. Much might be learned by tracking the trajectory of young children who enter the child welfare system due to physical abuse reported by a medical professional. But to do so, prenatal substance exposure would need to be clearly differentiated as a risk factor for physical abuse, not physical abuse in and of itself. While some states may adopt law or policy that considers prenatal substance exposure as de facto physical abuse, federal data should endeavor to separate the two.

### 4.3 Study limitations

This study is subject to several important limitations. NCANDS is an administrative dataset, not a population-based registry, and individuals cannot be reliably tracked across US states (11). While participation in this dataset is voluntary, currently all 50 states and DC participate. The exclusion of four states limits the external validity of this study as it does not represent the entire US. Generalizability is also limited to reports by medical professionals which are screened in by CPS agencies.

While we chose to exclude WV, MI, OH, and NV due to our concerns about their reporting practices, we likely have not eliminated reporting of IPSE as de facto physical abuse in the NCANDS dataset. This could mask any decreases in physical abuse over time. Child maltreatment types are defined primarily by state laws and policies, so physical abuse rates are likely to always vary between states (3). Inclusion of state-level policy effects may have strengthened our analysis and might be an avenue for future research in our population of interest. The SCAN database provides a valuable resource for such an effort in the future.

The large number of records included in our multivariable model is both a strength and a limitation. Weaker associations such as those for sex and race/ethnicity with substantiation may have arisen due to high power alone, though our use of an alpha level of 0.01 provided conservative estimates of the confidence intervals. The use of available data and extent of missing data limited our analysis by decreasing statistical power. Additionally, bias may have been introduced in the multivariable model because missingness of the COI covariate is not random: investigations in counties with small populations and those involving a fatality are suppressed, thus missing covariate COI). The results of our sensitivity analysis without a measure of SES had more complete data but yielded very similar results (Supplementary Table S4).

### 4.4 Conclusion

Children under 5 years of age, and especially infants, are at heightened risk of physical abuse which is often reported by a medical professional. Infants are a unique population who experience high risk of serious injury, foster care entry, or death as a result of physical abuse. This study suggests that previously-reported increases in investigations of physical abuse based on reports by medical professionals in recent years are related to misclassification of infant prenatal substance exposure as physical abuse in an important national child maltreatment dataset.

Nothing herein should be construed to indicate the support or endorsement of its content by the collector of the original data, their funding agency, NDACAN, or ACF/DHHS.

## Data Availability

The data analyzed in this study is subject to the following licenses/restrictions: This publication utilizes data from the NCANDS Child Files, 2014–2023, which have been provided by the National Data Archive on Child Abuse and Neglect (NDACAN), a service of the Children's Bureau, U.S. Department of Health & Human Services. Nothing herein should be construed to indicate the support or endorsement of its content by the collector of the original data, their funding agency, NDACAN, or ACF/DHHS. Requests to access these datasets should be directed to National Data Archive on Child Abuse and Neglect, https://www.ndacan.acf.hhs.gov/.

## References

[1] World Health Organizaion. Child maltreatment. (2024). Available online at: https://www.who.int/news-room/fact-sheets/detail/child-maltreatment (Accessed November 12, 2025).

[2] Gilbert R Fluke J O'Donnell M Gonzalez-Izquierdo A Brownell M Gulliver P Child maltreatment: variation in trends and policies in six developed countries. Lancet. (2012) 379(9817):758–72. 10.1016/S0140-6736(11)61087-8 22169108

[3] U.S. Department of Health & Human Services, Administration on Children, Children’s Bureau. Child maltreatment 2023 (2025). Available online at: https://www.acf.hhs.gov/cb/data-research/child-maltreatment (Accessed March 21, 2025).

[4] Edwards F Roberts SCM Kenny KS Raz M Lichtenstein M Terplan M. Medical professional reports and child welfare system infant investigations: an analysis of national child abuse and neglect data system data. Health Equity. (2023) 7(1):653–62. 10.1089/heq.2023.0136 37786528 PMC10541941

[5] Nadon M Park K Lee JY Wright M. Who makes the call? Examining the relationship between child maltreatment referral sources and case outcomes in the United States, 2008–2018. Child Abuse Negl. (2023) 145:106404. 10.1016/j.chiabu.2023.106404 37598611

[6] Comprehensive Addiction and Recovery Act of 2016. Pub. L. No. 114-198, 130 Stat. 695 (2016). Available online at: https://www.congress.gov/114/plaws/publ198/PLAW-114publ198.pdf (Accessed September 29, 2025).

[7] U.S. Department of Health & Human Services, Administration on Children, Children’s Bureau. FFY 2023 NDACAN dataset number 285 user’s guide. national child abuse and neglect data system (NCANDS) child file (2024).

[8] Gandarilla Ocampo M Drake B Simon J Jonson-Reid M. Does a child maltreatment report source predict differences in immediate and subsequent report outcomes? Child Abuse Negl. (2024) 147:106587. 10.1016/j.chiabu.2023.106587 38043457

[9] Foster RE Stone FP Linkh DJ Besetsny LK Collins PS Saha T Substantiation of spouse and child maltreatment reports as a function of referral source and maltreatment type. Mil Med. (2010) 175(8):560–6.10. 10.7205/MILMED-D-10-00035 20731259

[10] Children’s Bureau, Administration on Children, Youth and Families, Administration For Children And Families, U.S. Department Of Health And Human Services. Data from: national child abuse and neglect data system (NCANDS) child files (2014–2023). 10.34681/ekxj-by60

[11] Yi Y Wildeman C. How the AFCARS and NCANDS can provide insight into linked administrative data. In: Connell CM Crowley DM, editors. Strengthening Child Safety and Well-being through Integrated Data Solutions. Cham, Switzerland: Springer (2023). p. 13–31.

[12] US Census Bureau. Data from: Single-race Population Estimates, United States: Vintage 2020 Released July 27, 2021 for Years 2014–2019, Vintage 2023 Released June 27, 2024 for Years 2021–2023. Available online at: https://wonder.cdc.gov/wonder/help/single-race.html#About%202020-2023 (Accessed September 24, 2025).

[13] Noelke C McArdle N DeVoe B Leonardos M Lu Y Ressler RW Child opportunity index 3.0 technical documentation (2025). Available online at: diversitydatakids.org/research-library/coi-30-technical-documentation (Accessed September 25, 2025).

[14] Jones D Drake B Kim H Chen J-H Font S Putnam-Hornstein E Poverty indicators in the national child abuse and neglect data system child file: challenges and opportunities. Res Soc Work Pract. (2023) 34(3):325–37. 10.1177/10497315231179658

[15] Boston University School of Social Work: Institute for Equity in Child Opportunity & Healthy Development. Data from: child opportunity index 3.0 county data (2025). Available online at: diversitydatakids.org (Accessed October 01, 2025).

[16] R Core Team. R: A Language and Environment for Statistical Computing. Vienna, Austria: R Foundation for Statistical Computing (2016). Available online at: https://www.R-project.org/ (Accessed October 01, 2025).

[17] Bates D Mächler M Bolker B Walker S. Fitting linear mixed-effects models using lme4. J Stat Softw. (2015) 67(1):1–48. 10.18637/jss.v067.i01

[18] Wickham H. Ggplot2: Elegant Graphics for Data Analysis. Cham, Switzerland: Springer International Publishing (2016).

[19] Blangis F Drouin J Launay E Miranda S Zureik M Cohen JF Maternal, prenatal and postnatal risk factors for early child physical abuse: a French nationwide cohort study. Lancet Reg Health Eur. (2024) 42:100921. 10.1016/j.lanepe.2024.100921 39070743 PMC11281928

[20] Puls HT Berry JG Hall M. Trends in hospital encounters for child physical abuse through the COVID-19 pandemic. J Hosp Med. (2025) 20(12):1332–6. 10.1002/jhm.70056 40200574

[21] Shusterman GR Fluke JD Nunez JJ Fettig NB Kebede BK. Child maltreatment reporting during the initial weeks of COVID-19 in the US: findings from NCANDS. Child Abuse Negl. (2022) 134:105929. 10.1016/j.chiabu.2022.105929 36270070 PMC9556910

[22] Tonmyr L Li YA Williams G Scott D Jack SM. Patterns of reporting by health care and nonhealth care professionals to child protection services in Canada. Paediatr Child Health. (2010) 15(8):e25–32. 10.1093/pch/15.8.e25 21966240 PMC2952523

[23] Rebbe R Sieger ML Reddy J Prindle J. U.S. State rates of newborns reported to child protection at birth for prenatal substance exposure. Int J Drug Policy. (2024) 130:104527. 10.1016/j.drugpo.2024.104527 39059078 PMC11488208

[24] Mathematica. State child abuse and neglect policies database. Available online at: https://www.scanpoliciesdatabase.com/state-profiles (Accessed September 30, 2025).

[25] Cheng FY Beagan RA Grossman MR. Neonatal opioid withdrawal syndrome. The Pediatr Clin North Am. (2025) 72(4):639–59. 10.1016/j.pcl.2025.03.006 40619192

[26] McClaskey BR. A brief overview of neonatal abstinence syndrome. Neonatal Netw. (2025) 44(4):243–7. 10.1891/NN-2024-0057 40816757

[27] Goyal S Saunders KC Moore CS Fillo KT Ko JY Manning SE Identification of substance-exposed newborns and neonatal abstinence syndrome using ICD-10-CM—15 hospitals, Massachusetts, 2017. MMWR Morb Mortal Wkly Rep. (2020) 69(29):951–5. 10.15585/mmwr.mm6929a2 32701936 PMC7377822

[28] Font S Connell CM Goldstein EG Jones D. The intersection of prenatal substance exposure and child protection: evidence from Pennsylvania. J Policy Anal Manage. (2025) 45(1):e70049. 10.1002/pam.70049

