# Supplementary material for: Physical abuse of young children reported by medical professionals in the United States 2014–2023
Source: Front Pediatr. 2026 Jan 5;13:1718486. doi: 10.3389/fped.2025.1718486 (PMC12813114; doi:10.3389/fped.2025.1718486)
Supplement: Supplementary file 1 [file Datasheet1.pdf]

Supplementary Material

1 Supplementary Tables

Supplementary Table 1. Missingness of demographics, child and caregiver characteristics among children under 5 with an investigation for possible physical abuse reported by a medical profession, 2014-2023 in the United States (n=285,329)

Variable	Missing (n)	%
**Sex**	1,329	0.5
**Race/ethnicity**	32,590	11.4
**Urban/rural**	1,730	0.6
**COI quartile**	57,999	20.3
**Prior victim**	5,997	2.1
**Post-investigation services**	47,641	16.7
**Foster care services**	71,343	25
**Alcohol abuse**	116,893	41
**Drug abuse**	114,824	40.2
**Intellectual disability**	130,814	45.8
**Emotional disturbance**	128,068	44.9
**Visual/hearing impairment**	126,510	44.3
**Learning disability**	116,036	40.7
**Physical disability**	136,017	47.7
**Behavior problem**	124,890	43.8
**Other medical condition**	102,234	35.8
**IPSE**	54,775	37.4
**Parent alcohol abuse**	127,826	44.8
**Parent drug abuse**	125,018	43.8
**Parent intellectual disability**	156,995	55
**Parent emotional disturbance**	151,391	53.1
**Parent visual/hearing impairment**	161,133	56.5
**Parent learning disability**	150,677	52.8
**Parent physical disability**	156,252	54.8
**Parent medical condition**	148,012	51.9
**Domestic violence**	99,052	34.7
**Inadequate housing**	126,993	44.5
**Financial problems**	99,437	34.8
**Public assistance**	132,662	46.5

[a] Percentage of IPSE missingness is calculated out of the cohort with age < 1.
Percentages for all other variables were calculated out of the full cohort.

Supplementary Table 2. Median annual CPS investigation rates by medical professionals involving physical abuse in children < 5 years of age per 10,000 children, by State and Age Group (2014–2023)

	Age							
	**Overall**		**<1 year**		**1–2 years**		**3–4 years**	
**State or territory**	**Median**	**N**	**Median**	**N (%)**	**Median**	**N (%)**	**Median**	**N (%)**
Missouri	2.2	983	5.7	412 (41.9%)	2.2	321 (32.7%)	1.7	250 (25.4%)
Maryland	4.1	1790	11.7	828 (46.3%)	4.1	594 (33.2%)	2.5	368 (20.6%)
Montana	5.9	398	13.8	163 (41%)	5.9	141 (35.4%)	3.8	94 (23.6%)
New Jersey	6.4	4106	17.1	1740 (42.4%)	6.4	1331 (32.4%)	4.9	1035 (25.2%)
California	6.5	16266	13.0	5975 (36.7%)	6.5	6094 (37.5%)	4.4	4197 (25.8%)
Minnesota	7.2	3477	25.7	1724 (49.6%)	7.2	989 (28.4%)	5.4	764 (22%)
Wisconsin	7.2	2769	17.7	1126 (40.7%)	7.2	943 (34.1%)	5.2	700 (25.3%)
North Carolina	7.6	5458	19.2	2289 (41.9%)	7.6	1839 (33.7%)	5.4	1330 (24.4%)
Georgia	7.7	6148	23.3	2959 (48.1%)	7.7	1990 (32.4%)	4.5	1199 (19.5%)
Virginia	8.0	4898	23.0	2276 (46.5%)	8.0	1608 (32.8%)	5.0	1014 (20.7%)
Washington	8.2	3766	14.6	1264 (33.6%)	8.2	1454 (38.6%)	5.8	1048 (27.8%)
North Dakota	8.6	561	25.0	264 (47.1%)	8.6	182 (32.4%)	5.5	115 (20.5%)
Wyoming	8.7	323	17.9	121 (37.5%)	8.7	120 (37.2%)	5.7	82 (25.4%)
Colorado	8.7	3187	21.4	1370 (43%)	8.7	1123 (35.2%)	5.2	694 (21.8%)
Massachusetts	8.7	3830	19.6	1384 (36.1%)	8.7	1230 (32.1%)	8.4	1216 (31.7%)
New York	9.6	11522	14.8	3341 (29%)	9.6	4336 (37.6%)	8.5	3845 (33.4%)
Connecticut	9.8	2124	29.1	1025 (48.3%)	9.8	710 (33.4%)	5.2	389 (18.3%)
Arizona	10.0	4796	26.8	2179 (45.4%)	10.0	1664 (34.7%)	5.5	953 (19.9%)
New Mexico	10.6	1375	24.6	571 (41.5%)	10.6	509 (37%)	5.9	295 (21.5%)
Iowa	11.7	2459	24.5	928 (37.7%)	11.7	905 (36.8%)	7.9	626 (25.5%)
Florida	12.1	14830	27.9	6145 (41.4%)	12.1	5365 (36.2%)	7.3	3320 (22.4%)
Oregon	12.4	2914	23.4	1004 (34.5%)	12.4	1100 (37.7%)	8.8	810 (27.8%)
Pennsylvania	13.0	9755	28.0	3800 (39%)	13.0	3602 (36.9%)	8.3	2353 (24.1%)
Louisiana	13.4	4432	31.7	1886 (42.6%)	13.4	1611 (36.3%)	7.7	935 (21.1%)
Arkansas	13.5	2890	26.3	963 (33.3%)	13.5	1007 (34.8%)	12.1	920 (31.8%)

Oklahoma	14.3	4551	40.4	2010 (44.2%)	14.3	1452 (31.9%)	10.4	1089 (23.9%)
New Hampshire	14.3	991	26.1	319 (32.2%)	14.3	361 (36.4%)	11.8	311 (31.4%)
Hawaii	15.3	465	15.3	260 (55.9%)	4.1	142 (30.5%)	1.8	63 (13.5%)
Illinois	15.6	12832	34.2	4923 (38.4%)	15.6	4596 (35.8%)	10.9	3313 (25.8%)
Kansas	15.9	3159	31.1	1128 (35.7%)	15.9	1178 (37.3%)	11.1	853 (27%)
South Dakota	15.9	359	15.9	190 (52.9%)	4.5	109 (30.4%)	2.5	60 (16.7%)
Kentucky	16.5	4802	34.4	1844 (38.4%)	16.5	1793 (37.3%)	10.6	1165 (24.3%)
DC	16.6	2632	34.0	1349 (51.3%)	16.6	840 (31.9%)	13.5	443 (16.8%)
Utah	16.9	4876	42.3	2046 (42%)	16.9	1651 (33.9%)	11.8	1179 (24.2%)
Alaska	22.4	1337	57.0	584 (43.7%)	22.4	458 (34.3%)	14.3	295 (22.1%)
Delaware	24.6	1469	45.2	486 (33.1%)	24.6	536 (36.5%)	20.1	447 (30.4%)
Maine	27.9	2329	93.4	1141 (49%)	27.9	704 (30.2%)	18.5	484 (20.8%)
Indiana	29.4	12641	59.9	4848 (38.4%)	29.4	4866 (38.5%)	17.2	2927 (23.2%)
Vermont	42.9	1369	90.6	508 (37.1%)	42.9	498 (36.4%)	30.1	363 (26.5%)
Idaho	45.2	1955	45.2	1003 (51.3%)	11.7	531 (27.2%)	9.0	421 (21.5%)
Texas	80.0	49644	80.0	30749 (61.9%)	14.4	11273 (22.7%)	9.5	7622 (15.4%)
Mississippi	83.6	4593	83.6	3021 (65.8%)	12.7	927 (20.2%)	8.7	645 (14%)
Nebraska	99.4	3219	99.4	2524 (78.4%)	8.7	450 (14%)	4.7	245 (7.6%)
Alabama	152.3	12134	152.3	8816 (72.7%)	17.0	1989 (16.4%)	11.1	1329 (11%)
Rhode Island	185.4	2190	185.4	1953 (89.2%)	5.5	118 (5.4%)	5.4	119 (5.4%)
South Carolina	202.3	16160	202.3	11460 (70.9%)	23.4	2699 (16.7%)	16.9	2001 (12.4%)
Nevada	230.2	8830	230.2	8007 (90.7%)	6.7	478 (5.4%)	4.7	345 (3.9%)
Tennessee	243.9	26565	243.9	19619 (73.9%)	25.5	4147 (15.6%)	17.0	2799 (10.5%)
Ohio	311.0	53419	311.0	41546 (77.8%)	25.0	6845 (12.8%)	18.0	5028 (9.4%)
Michigan	395.4	48421	395.4	42976 (88.8%)	14.0	3126 (6.5%)	10.1	2319 (4.8%)
West Virginia	633.3	17091	633.3	11560 (67.6%)	78.9	2970 (17.4%)	66.0	2561 (15%)

Supplementary Table 3. Child and caregiver characteristics by age group

Risk Factor		Overall N = 285,329	<1 year N = 146,518 (51%)	1-2 years N = 82,086 (29%)	3-4 years N = 56,725 (20%)
**Child**					
**Alcohol abuse[ab]**	No	166,114 (99%)	87,691 (98%)	46,878 (100%)	31,545 (100%)
	Yes	2,322 (1.4%)	2,028 (2.3%)	177 (0.4%)	117 (0.4%)
**Drug abuse[ab]**	No	150,514 (88%)	72,968 (80%)	46,276 (98%)	31,270 (99%)
	Yes	19,991 (12%)	18,799 (20%)	819 (1.7%)	373 (1.2%)
**IPSE[b]**	No	72,396 (79%)	72,396 (79%)	0 (NA%)	0 (NA%)
	Yes	19,347 (21%)	19,347 (21%)	0 (NA%)	0 (NA%)
**Intellectual disability[ab]**	No	154,014 (100%)	86,278 (100%)	40,380 (100%)	27,356 (99%)
	Yes	501 (0.3%)	130 (0.2%)	176 (0.4%)	195 (0.7%)
**Emotional disturbance[ab]**	No	156,827 (100%)	87,715 (100%)	41,191 (100%)	27,921 (99%)
	Yes	434 (0.3%)	90 (0.1%)	127 (0.3%)	217 (0.8%)
**Visual/hearing impariment[ab]**	No	158,250 (100%)	87,853 (100%)	41,990 (100%)	28,407 (99%)
	Yes	569 (0.4%)	278 (0.3%)	141 (0.3%)	150 (0.5%)
**Learning disability[ab]**	No	168,696 (100%)	95,139 (100%)	43,903 (100%)	29,654 (99%)
	Yes	597 (0.4%)	74 (<0.1%)	183 (0.4%)	340 (1.1%)
**Physical disability[ab]**	No	148,480 (99%)	80,404 (100%)	40,580 (99%)	27,496 (99%)
	Yes	832 (0.6%)	360 (0.4%)	246 (0.6%)	226 (0.8%)
**Behavior problem[ab]**	No	158,956 (99%)	85,932 (100%)	44,089 (99%)	28,935 (98%)
	Yes	1,483 (0.9%)	355 (0.4%)	439 (1.0%)	689 (2.3%)
**Other medical condition[ab]**	No	164,230 (90%)	91,968 (89%)	43,247 (91%)	29,015 (91%)
	Yes	18,865 (10%)	11,863 (11%)	4,100 (8.7%)	2,902 (9.1%)
**Caregiver or family**					
**Alcohol abuse[ab]**	No	152,030 (97%)	79,581 (96%)	43,210 (97%)	29,239 (97%)
	Yes	5,473 (3.5%)	3,152 (3.8%)	1,315 (3.0%)	1,006 (3.3%)
**Drug abuse[ab]**	No	129,446 (81%)	61,078 (72%)	40,688 (91%)	27,680 (91%)
	Yes	30,865 (19%)	23,840 (28%)	4,170 (9.3%)	2,855 (9.3%)

**Intellectual disability[ab]**	No	127,028 (99%)	68,120 (99%)	35,255 (99%)	23,653 (99%)
	Yes	1,306 (1.0%)	707 (1.0%)	334 (0.9%)	265 (1.1%)
**Emotional disturbance[ab]**	No	126,604 (95%)	68,185 (94%)	35,006 (95%)	23,413 (94%)
	Yes	7,334 (5.5%)	4,115 (5.7%)	1,848 (5.0%)	1,371 (5.5%)
**Visual/hearing impairment[ab]**	No	123,691 (100%)	67,035 (100%)	33,894 (100%)	22,762 (100%)
	Yes	505 (0.4%)	258 (0.4%)	138 (0.4%)	109 (0.5%)
**Learning disability[ab]**	No	132,829 (99%)	73,090 (99%)	35,708 (99%)	24,031 (99%)
	Yes	1,823 (1.4%)	1,105 (1.5%)	428 (1.2%)	290 (1.2%)
**Physical disability[ab]**	No	128,113 (99%)	68,717 (99%)	35,568 (99%)	23,828 (99%)
	Yes	964 (0.7%)	502 (0.7%)	268 (0.7%)	194 (0.8%)
**Other medical condition[ab]**	No	134,482 (98%)	73,699 (98%)	36,341 (98%)	24,442 (98%)
	Yes	2,835 (2.1%)	1,648 (2.2%)	703 (1.9%)	484 (1.9%)
**Domestic violence[ab]**	No	171,717 (92%)	91,611 (93%)	47,479 (91%)	32,627 (91%)
	Yes	14,560 (7.8%)	6,852 (7.0%)	4,421 (8.5%)	3,287 (9.2%)
**Inadequate housing[ab]**	No	151,648 (96%)	78,683 (95%)	43,096 (97%)	29,869 (97%)
	Yes	6,688 (4.2%)	4,310 (5.2%)	1,472 (3.3%)	906 (2.9%)
**Financial problems[ab]**	No	172,931 (93%)	96,652 (93%)	45,331 (93%)	30,948 (93%)
	Yes	12,961 (7.0%)	7,141 (6.9%)	3,548 (7.3%)	2,272 (6.8%)
**Public assistance[ab]**	No	123,718 (81%)	68,476 (80%)	32,944 (83%)	22,298 (82%)
	Yes	28,949 (19%)	17,255 (20%)	6,912 (17%)	4,782 (18%)

[a] Pearson's Chi-squared test <0.01
[b] Percentages do not include missing data
IPSE: Infant with Prenatal Substance Exposure

Supplementary Table 4. Sensitivity analysis: removing COI from multivariable analysis of adjusted odds of substantiation of a physical abuse report by a medical professional from 2014-2023 in children under 5 years of age.

Variable (fixed effects)		aOR	99% CI
Age			
	1-4 years (Ref)		
	<1 year	2.65	2.58, 2.72
Sex			
	Female (Ref)		
	Male	1.05	1.03, 1.08
Race/ethnicity			
	NH White (Ref)		
	NH Black	1.13	1.09, 1.16
	Hispanic	0.97	0.94, 1.00
	NH Asian	0.65	0.58, 0.73
	NH AIAN/NHPI	1.14	1.03, 1.25
Neglect also on report		1.45	1.41, 1.49
Prior victim		1.33	1.29, 1.37

Includes 46 US states and the District of Columbia, excludes West Virginia, Michigan, Ohio and Nevada; N=246,101, groups=47
Random effects: Adjusted ICC=0.170
AIC=270980.1

STROBE Statement—Checklist of items that should be included in reports of *cohort studies*

	Item No	Recommendation
**Title and abstract**	1	(*a*) Indicate the study's design with a commonly used term in the title or the abstract
		(*b*) Provide in the abstract an informative and balanced summary of what was done and what was found
**Introduction**		
Background/rationale	2	Explain the scientific background and rationale for the investigation being reported
Objectives	3	State specific objectives, including any prespecified hypotheses
**Methods**		
Study design	4	Present key elements of study design early in the paper
Setting	5	Describe the setting, locations, and relevant dates, including periods of recruitment, exposure, follow-up, and data collection
Participants	6	(*a*) Give the eligibility criteria, and the sources and methods of selection of participants. Describe methods of follow-up
		(*b*) For matched studies, give matching criteria and number of exposed and unexposed
Variables	7	Clearly define all outcomes, exposures, predictors, potential confounders, and effect modifiers. Give diagnostic criteria, if applicable
Data sources/ measurement	8*	For each variable of interest, give sources of data and details of methods of assessment (measurement). Describe comparability of assessment methods if there is more than one group
Bias	9	Describe any efforts to address potential sources of bias
Study size	10	Explain how the study size was arrived at
Quantitative variables	11	Explain how quantitative variables were handled in the analyses. If applicable, describe which groupings were chosen and why
Statistical methods	12	(*a*) Describe all statistical methods, including those used to control for confounding
		(*b*) Describe any methods used to examine subgroups and interactions
		(*c*) Explain how missing data were addressed
		(*d*) If applicable, explain how loss to follow-up was addressed
		(*e*) Describe any sensitivity analyses
**Results**		
Participants	13*	(a) Report numbers of individuals at each stage of study—eg numbers potentially eligible, examined for eligibility, confirmed eligible, included in the study, completing follow-up, and analysed
		(b) Give reasons for non-participation at each stage
		(c) Consider use of a flow diagram
Descriptive data	14*	(a) Give characteristics of study participants (eg demographic, clinical, social) and information on exposures and potential confounders
		(b) Indicate number of participants with missing data for each variable of interest
		(c) Summarise follow-up time (eg, average and total amount)
Outcome data	15*	Report numbers of outcome events or summary measures over time
Main results	16	(*a*) Give unadjusted estimates and, if applicable, confounder-adjusted estimates and their precision (eg, 95% confidence interval). Make clear which confounders were adjusted for and why they were included
		(*b*) Report category boundaries when continuous variables were categorized
		(*c*) If relevant, consider translating estimates of relative risk into absolute risk for a meaningful time period
Other analyses	17	Report other analyses done—eg analyses of subgroups and interactions, and sensitivity analyses
**Discussion**		
Key results	18	Summarise key results with reference to study objectives
Limitations	19	Discuss limitations of the study, taking into account sources of potential bias or imprecision. Discuss both direction and magnitude of any potential bias

Interpretation	20	Give a cautious overall interpretation of results considering objectives, limitations, multiplicity of analyses, results from similar studies, and other relevant evidence
Generalisability	21	Discuss the generalisability (external validity) of the study results
**Other information**		
Funding	22	Give the source of funding and the role of the funders for the present study and, if applicable, for the original study on which the present article is based

*Give information separately for exposed and unexposed groups.

Note: An Explanation and Elaboration article discusses each checklist item and gives methodological background and published examples of transparent reporting. The STROBE checklist is best used in conjunction with this article (freely available on the Web sites of PLoS Medicine at http://www.plosmedicine.org/, Annals of Internal Medicine at http://www.annals.org/, and Epidemiology at http://www.epidem.com/). Information on the STROBE Initiative is available at http://www.strobe-statement.org.
